# Patients’ Perspectives about Lifestyle Behaviors and Health in the Context of Family Medicine: A Cross-Sectional Study in Portugal

**DOI:** 10.3390/ijerph18062981

**Published:** 2021-03-14

**Authors:** Rosália Páscoa, Andreia Teixeira, Micaela Gregório, Rosa Carvalho, Carlos Martins

**Affiliations:** 1 Department of Community Medicine, Information and Health Decision Sciences (MEDCIDS), Faculty of Medicine, University of Porto, 4200-450 Porto, Portugal; andreiasofiat@med.up.pt (A.T.); micaelagregorio96@gmail.com (M.G.); carlosmartins20@gmail.com (C.M.); 2 Centre for Health Technology and Services Research (CINTESIS), Faculty of Medicine, University of Porto, 4200-450 Porto, Portugal; 3 Instituto Politécnico de Viana do Castelo (IPVC), 4900-347 Viana do Castelo, Portugal; 4 #H4A Primary Healthcare Research Network, 4460-027 Porto, Portugal; ocorreiodarosa@gmail.com

**Keywords:** lifestyle, family doctor, behavior, chronic/non-communicable diseases

## Abstract

Lifestyle interventions are recognized as essential in the prevention and treatment of non-communicable diseases. Previous studies have shown that Portuguese patients tend to give more importance to diagnostic and laboratory tests than to lifestyle measures, and seem unaware that behavioral risks are the main modifiable risk factors. The study aimed to analyze patients’ perspectives about lifestyle behaviors and health in the context of family medicine in Portugal. A population-based cross-sectional study was carried out in Portugal (the mainland). A total of 900 Portuguese patients aged ≥20 years, representative of the population, were surveyed using face-to-face questionnaires. Participants were selected by the random route method. Descriptive statistics and non-parametric tests were performed to evaluate differences between the personal beliefs and the personal behavior self-assessment, as well as between the level of importance given to the family doctor to address health behaviors and the reported approach implemented by the family doctor, and its association with bio-demographic variables. The results indicate that the vast majority of this Portuguese cohort has informed beliefs regarding lifestyle behaviors, tends to overestimate their own behavior self-assessment, and strongly agrees that it is important that their family doctor asks/advises on these lifestyle behaviors, although the proportion of those who totally agree that their family doctor usually does this is significantly lower. Differences concerning bio-demographic variables were found. Future research directions should focus on the politics, economics, and policy aspects that may have an impact in this area. It will also be important to understand more broadly the relationships between lifestyle behaviors and clinical, physical, and sociodemographic variables.

## 1. Introduction

Non-communicable diseases account for 80% of deaths in the European region and 70% of global deaths [1,2], representing a worldwide burden and a major public health challenge [3]. Health-promoting lifestyle patterns are a strategic key [3], as healthy behaviors can positively influence the outcomes of chronic diseases [4,5]. In this regard, primary healthcare is seen as a priority in the strengthening of health systems [6,7]. Family medicine discipline characteristics indicate that this is usually the point of first medical contact in the health system, and it is important to: developing a person-centred approach, promoting patient empowerment, providing longitudinal continuity care, managing chronic problems, and promoting health and well-being [8]. These characteristics also place family medicine in a privileged position to deal with lifestyle behaviors. 

Tobacco use, physical inactivity, harmful consumption of alcohol, and unhealthy diet are modifiable behaviors recognized as major risk factors for non-communicable diseases, constituting important targets of action [2,3,4]. Sedentary lifestyle, stress, and sleep patterns have also been identified as chronic disease modifiers [9,10]. Lifestyle interventions play an important role in the primary, secondary, and tertiary preventions of chronic diseases, including diabetes, heart disease, stroke, cancer, and chronic lung disease [11,12,13,14,15,16,17,18]. The benefits of a healthy lifestyle have been noted by large-scale epidemiological studies [19,20,21,22], systematic reviews, and meta-analyses [23,24,25,26]. According to these, the guidelines of several scientific societies advocate lifestyle interventions as first-line and alongside for the prevention and treatment of these common diseases [27,28,29,30,31,32,33]. From a practical point of view, taking into account the necessary operationalization of this type of intervention, the research carried out by the DEDIPAC project stands out, which identified characteristics of interventions and policies that promote healthy eating, physical activity, and reduction of sedentary behavior [34].

Potential non-negligible effects of behavior change on mortality, morbidity, and healthcare costs contributed to the concept of lifestyle medicine (i.e., evidence-based practice supporting individuals and families to adopt and sustain behaviors that can improve health and quality of life) [4]. The absence or quasi-absence of iatrogenic effects of lifestyle interventions is one of the attractive aspects [35]. 

A previous study has shown that Portuguese patients view diagnostic and laboratory tests as more important than lifestyle measures, and seem unaware that behavioral risks are the main modifiable risk factors [36]. This study is pertinent in better understanding the patient’s beliefs and perspectives about lifestyle interventions, including diet, physical activity, alcohol consumption, tobacco use, illicit drugs, sleep habits, screen activities, stress, and being sedentary. To the best of the authors’ knowledge, this is the first time that a study on lifestyle behaviors and health has been carried out in the Portuguese context. Furthermore, this study is innovative in trying to understand the opinion on family doctors in addressing lifestyle behaviors. Thus, the cohort of the study was approached on four main aspects, which will be designated by dimensions. Dimension 1: personal belief about each lifestyle intervention (can prevent and help control vs. can cause and aggravate some diseases, as appropriate); dimension 2: personal behavior self-assessment (if, in the personal case, each lifestyle intervention is healthy or not); dimension 3: level of importance given to the family doctor to address each lifestyle intervention; and dimension 4: if, in the personal case, something was usual or not, the family doctor asks/advises on each lifestyle intervention.

The aims of this study were twofold: (i) centered around lifestyle behaviors and (ii) centered around family doctors in these behaviors. The hypotheses (H) raised by the authors were as follows:

**Hypothesis** **1** **(H1).** *The Portuguese personal beliefs about lifestyle in the prevention and treatment of some diseases (dimension 1) are in accordance with the self-assessment of personal behavior, for each lifestyle intervention (dimension 2).*

**Hypothesis** **2** **(H2).** *The level of importance given to the family doctor to address lifestyle interventions (dimension 3) is in line with the reported approach implemented by the family doctor (dimension 4) for each lifestyle intervention.*

**Hypothesis** **3** **(H3).** *The responses of Portuguese patients (about the four dimensions) are different regarding gender, age, marital status, education level, general health status, and the number of problems in the last 12 months.*

## 2. Materials and Methods

### 2.1. Study Design and Participants

A cross-sectional, population-wide study was conducted in a representative sample of the Portuguese adult general population, aged 20 years or over, using a face-to-face interview. Exclusion criteria included having a cognitive or physical disability that hampered the ability to participate in a face-to-face interview, being a resident of a collective dwelling, not speaking/understanding Portuguese, and refusal to give informed consent for study participation. 

To obtain a representative sample of the Portuguese general adult population, a stratified sampling design was used. First, all NUTS II (nomenclature of territorial units for statistical purposes) were used as natural strata; in each NUTS II, a random sample of starting points was selected with a probability proportional to the NUTS population size, as estimated by the national census [37]. Target quotas were set considering the distribution of the variables gender (male; female), age (groups every five years, except the last defined group: 20–24, 25–29, 30–34, 35–39, 40–44, 45–49, 50–54, 55–59, 60–64, 65–69, 70–74, and ≥75), and region of residence (North, Center, Lisbon, Alentejo, and Algarve). Given the geographical dispersion, interviews were conducted in all district capitals, ensuring the proportionality that they represent in the resident population of mainland Portugal.

A sample size of 900 participants was calculated for a 95% confidence level, considering the most conservative scenario (*p* = 0.5), an infinite population, and a margin of error of approximately 3%. 

Participants were selected by application of the random route sampling method [38], which implied that each interviewer had an interview number and quotas to reach. The daily visit plan was defined based on a totally random choice of the street, door number, and floor, called the starting point. Individuals, one in each household, were selected using the last birthday method (selection in each home, and on the date of the interview, of the person residing in the household who celebrated his/her birthday more recently). If the quota of the identified individual was fulfilled or the individual did not agree to participate in the study, the previous birthday would be identified. This was done in the same way, considering the order of the date of the last birthday, until the individuals residing in the selected household were exhausted. If no response was obtained in an address, three new contacts were made at different days and times. If there was no response (or no element could be selected in an address), it would be replaced by another address following the rules of the random route method. To identify as many people as possible at home, fieldwork was preferably carried out from 5 pm to 9 pm on weekdays, and from 11 am to 9 pm on weekends and holidays.

### 2.2. Data Collection

The data collection was performed from 16 January to 30 April 2019 using a questionnaire (Supplementary Materials: File S1) in Portuguese language. The questionnaire was specifically designed for this study by the authors, after researching previous studies available [39,40]. Completion of the questionnaire took between 20 to 25 min following the order of the questions; in each questionnaire, the responses were recorded manually. 

A pre-test of 20 interviews was carried out to assess language issues, comprehension of the questions, and time required for the application of the questionnaire. Next, the investigation team met with some of the interviewers to listen to their feedback. No changes were required to the questionnaire, but these 20 interviews were not included in the final sample. All of the participants involved in the pilot testing were Portuguese and from the northern part of the country. The mean age was 47.1 ± 17.1 years (23–85 years), 55% (*n* = 11) were female, and 70% (*n* = 14) were married. Among these participants, 35% (*n* = 7) had completed primary school, approximately 55% (*n* = 11) were employed by others, and 95% (*n* = 19) worked in the tertiary professional sector. Private health insurance was not reported by anyone (Supplementary Materials: Table S1).

The interviewers were trained to clarify the meaning of each question to ensure that participants correctly understood all of the questions, and were trained to do standard operating procedures for all contacts made. For quality control, all interviews were monitored by a data collection supervisor, and at least 20% were randomly supervised by members of the investigation team. 

A structured questionnaire containing three sections, after an introductory section presenting study aims and motivation, was used: (1) questions about health status; (2) main research section; and (3) sociodemographic data. In the main research section, the interviewees answered: (A) a Likert scale (1 = strongly disagree, 2 = partially disagree, 3 = indifferent, 4 = partially agree, and 5 = strongly agree) on different lifestyle interventions (diet, physical activity, alcohol consumption, tobacco use, illicit drugs, sleep habits, screen, stress, and being sedentary); and (B) nine closed and direct questions about their pattern of lifestyle, including all of the different interventions identified above. In the Likert scale, respondents reported for each lifestyle intervention: (1) personal belief about each lifestyle intervention (can prevent and help control vs. can cause and aggravate some diseases, as appropriate)—dimension 1; (2) personal behavior self-assessment (if, in the personal case, each lifestyle intervention is healthy or not)—dimension 2; (3) level of importance given to the family doctor to address each lifestyle intervention—dimension 3; and (4) if, in the personal case, something was usual or not, the family doctor asks/advises on each lifestyle intervention—dimension 4.

### 2.3. Definition of the Variables of Lifestyle

During the interview, lifestyle pattern, including the interventions referred, was recorded in detail. For the intended analysis, the authors created new variables with some of the collected data through the questions about lifestyle pattern in the main research section.

#### 2.3.1. Diet

A healthy diet was considered whenever the participant concomitantly ate two to three main meals per day, two to six portions of fruit per day, two or more portions of legumes or salads per day, and had a moderate consumption of alcohol (defined below) [41,42,43].

#### 2.3.2. Physical Activity

Regular physical activity was defined as walking or doing any physical activity for five or more days of the week and for at least 30 to 59 min on average [44]. 

#### 2.3.3. Alcohol Consumption

Moderate alcohol consumption was defined as a maximum of one drink per day in the case of females, or males 65 years or older. For males under 65 years old, it was considered a maximum of two drinks per day [45]. 

#### 2.3.4. Tobacco Use

The correct use of tobacco was considered when participants answered “No” to the question “Do you smoke?” [46]. 

#### 2.3.5. Illicit Drugs

The correct use of illicit drugs was considered when participants answered “No” to the question “Do you use illicit drugs?” [47]. 

#### 2.3.6. Sleep Habits

Good quality sleep was considered when participants simultaneously selected “few days or never” for the frequency of sleep problems (difficulty falling asleep, slept poorly, or overslept), as well as for the need to take medication to sleep, and answered “most days or always” for having a repairing sleep in the last two weeks [48,49].

#### 2.3.7. Screen Activities

Moderate screen activities were defined as, on a normal day, having had a screen time of fewer than 3 h [50,51].

#### 2.3.8. Stress

The moderate stress level was considered when participants answered, simultaneously, “few days or never” about doing less than they wanted in work/daily activities for feeling anxious/nervous, as well as for the frequency of feeling anxious/nervous, and answered “most days or always” for the need for medication to control anxiety and nervousness and feel calm in the last two weeks [52].

#### 2.3.9. Being Sedentary

Participants were considered not sedentary when the option that best described their main daily activities (working, taking care of the house, taking care of family members, studying, volunteering…) was “on the move or on tasks that require moderate physical effort” or “in heavy or physically demanding jobs” [53].

### 2.4. Statistical Analysis

Statistical analysis was performed using Microsoft Excel 2016^®^ and the Statistical Package for the Social Sciences (SPSS^®^) version 25.0 for Windows^®^. Descriptive statistics were represented as absolute frequency (*n*) and relative frequency (%) for categorical variables. Continuous variables were described by mean and standard deviation, $\bar{x}\pm \mathrm{sd}$, and by the minimum and maximum (min-max) values. Ordinal variables were presented by the median (Med) and respective 95% confidence interval (95% CI) or the respective interquartile interval [Q_1_; Q_3_], where Q_1_ is the first quartile and Q_3_ is the third quartile. 

To compare related ordinal variables (the personal beliefs—dimension 1 and the personal behavior self-assessment—dimension 2, as well as the level of importance given to the family doctor to address health behaviors—dimension 3 and the reported approach implemented by the family doctor—dimension 4), the Wilcoxon test was used and dumbbell charts were constructed to visualize the difference between the median values of ordinal variables. For each of the four dimensions (ordinal variables), its association with bio-demographic variables was studied. For gender, marital status, and education level, the Mann–Whitney test was used. For the number of problems in the last 12 months, the Kruskal–Wallis test was performed, and when statistical differences were found, multiple comparisons with Bonferroni adjustments were calculated. For the number of health problems and age, the Spearman coefficient and the respective *p*-value were used. The internal consistency of the main section of the questionnaire was assessed using Cronbach’s alpha (α); *p*-values ≤ 0.05 were considered significant.

## 3. Results

During face-to-face questionnaires, 25 individuals were excluded for not speaking/ understanding Portuguese and 198 individuals refused to participate, which meant a response rate of about 82%.

Nine-hundred participants answered the questionnaire, the sample was representative of the general adult Portuguese population, and the relevant sample characteristics are presented in Table 1. To compare our sample with the Portuguese population, we produced a table (Supplementary Materials: Table S2) with the sociodemographic data of the population that could be obtained from the PORDATA website (Accessed on 2 July 2019, www.pordata.pt).

Table 2 shows the distribution of the participants according to the perception of the general health status and according to the reported health problems.

The Likert scale responses about the level of agreement with statements on lifestyle and lifestyle in the context of family medicine presented a good internal consistency for (dimension 1) belief about lifestyle interventions (α = 0.867), for dimension 3, level of importance given to the family doctor to address health behaviors (α = 0.882), and dimension 4 for if in the personal case was usual or not that the family doctor asks/advises about that health behavior (α = 0.901). Cronbach’s α of the personal behavior self-assessment (dimension 2) was 0.549. 

The analysis of this part of the main section of the questionnaire is shown in Table 3, and demonstrated that the median of the level of importance attributed to lifestyle interventions in the prevention and control of some diseases is maximum for all lifestyle interventions studied. Regarding the personal behavior self-assessment, the area of consumption (illicit drugs, tobacco, and alcohol) is the one that stands out as being the most reported as correctly fulfilled. Participants attributed higher levels of importance to the advice from the family doctor about diet, sleep habits, stress, and being sedentary. From the participants’ point of view, the family doctor most often asks/advises on diet.

**Figure 1:**
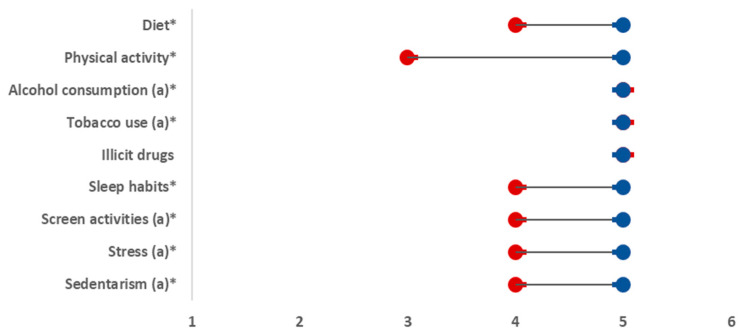
The differences between personal beliefs (dimension 1) and personal behavior self-assessment (dimension 2) for each lifestyle intervention. (*) Significant at 5% (Wilcoxon test with Bonferroni adjustment), *p*-value < 0.001. (a) Inversion was performed to facilitate the comparison between the variables. Red = median value of personal behavior self-assessment. Blue = median value of personal belief. Note: a higher value is better than a lower value. The only exception existed for the behaviors marked with (a), for which an inversion was carried out to facilitate the comparison.

**Figure 2:**
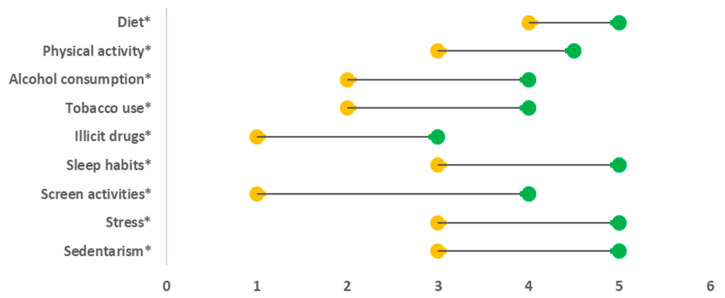
The differences between level of importance given to the family doctor to address health behaviors (dimension 3) and the reported approach implemented by the family doctor (dimension 4) for each lifestyle intervention. (*) Significant at 5% (Wilcoxon test with Bonferroni adjustment). Yellow = median value of the variable if, in the personal case, something was usual or not, the family doctor asks/advises. Green = median value of the variable level of importance given to the family doctor to address. Note: a higher value is better than a lower value.

To verify the hypotheses H_1_ and H_2_, the Wilcoxon test was performed. Significant differences were detected between the personal beliefs (dimension 1) and the personal behavior self-assessment (dimension 2), as well as between the level of importance given to the family doctor to address health behaviors (dimension 3) and the reported approach implemented by the family doctor (dimension 4) for each lifestyle intervention (Figure 1 and Figure 2). More detailed information is presented in Supplementary Materials: Table S3.

The general characterization of the lifestyle pattern of participants, obtained through the answers to the nine questions on the main section of the questionnaire, are shown in Table 4. More detailed information is presented in Supplementary Materials, Table S4.

Regarding the hypothesis H_3_ raised by the authors: “the responses of Portuguese patients are different regarding gender, age, marital status, education level, general health status, and the number of problems in the last 12 months,” the association with bio-demographic variables was studied for each lifestyle intervention regarding the four dimensions. 

The results are presented in Table 5 in more detail, and show that, for dimension 1 (personal belief about each lifestyle intervention), the personal belief was particularly different according the number of health problems in the last 12 months (alcohol consumption, tobacco use, and illicit drugs) and age (physical activity). 

For dimension 2 (if, in the personal case, each lifestyle intervention is healthy or not), the personal behavior self-assessment was different according to the number of health problems in the last 12 months (diet, physical activity, alcohol consumption, tobacco use, illicit drugs, sleep habits, stress, and being sedentary), age (physical activity, alcohol consumption, tobacco use, sleep habits, screen activities, stress, and being sedentary), general health status (diet, physical activity, tobacco use, sleep habits, and being sedentary), gender (alcohol consumption, tobacco use, sleep habits, and stress), education level (physical activity, sleep habits, screen activities, and stress), and marital status (physical activity and tobacco use).

Regarding dimension 3 (level of importance given to the family doctor to address each lifestyle intervention), differences were found for general health status (diet, physical activity, alcohol consumption, tobacco use, sleep habits, and screen activities), age (diet, tobacco use, illicit drugs, sleep habits, and screen activities), the number of health problems (tobacco use, illicit drugs, and screen activities), gender (physical activity), marital status (screen activities), and educational level (illicit drugs). 

Dimension 4 (if, in the personal case, something was usual or not, the family doctor asks/advises on each lifestyle intervention) was different regarding age (diet, tobacco use, illicit drugs, sleep habits, stress, and being sedentary), general health status (alcohol consumption, screen activities, stress, and being sedentary), the number of health problems in the last 12 months (alcohol consumption, tobacco use, illicit drugs, and screen activities), gender (alcohol consumption, tobacco use, and sleep habits), marital status (diet), and education level (sleep habits).

## 4. Discussion

This study shows that the vast majority of this Portuguese cohort has informed beliefs regarding main lifestyle behaviors. They believe that a healthy diet, regular physical activity, and good quality sleep can prevent and help control some diseases, and they believe that smoking tobacco, taking illicit drugs, excessive screen time, excessive stress, and being sedentary can cause or aggravate some diseases. However, they appear to have some difficulties in identifying the failures in their lifestyle factors (Figure 1), and, in addition, the scientific recommendations on these issues. Another major conclusion is that the proportion of participants who strongly agree that a family doctor’s approach on lifestyle (dimension 3) is important is much higher than the proportion of those who totally agree that their family doctor usually does this (dimension 4) (Figure 2).

Our results suggest that participants overestimate their own behavior during self-assessment, a situation described in previous studies [54,55]. Although 87.0% (*n* = 783) totally agreed, and 12% (*n* = 108) partially agreed with “my diet is healthy”, only 14% (*n* = 126) of participants met the healthy diet criteria defined by the authors. Similarly, through the auto-reported values of weight and height, 40.2% (*n* = 358) of participants presented as overweight and 13.4% (*n* = 119) as obese, but the self-reported prevalence of being overweight and obese was 15.6% (*n* = 140) and 2.1% (*n* = 19), respectively (Table 2). Regarding regular physical activity, 30.1% (*n* = 271) totally agreed and 18.7% (*n* = 168) partially agreed with “my physical activity is regular”, but only 28.9% (*n* = 260) achieved the criteria of regular physical activity. Concerning alcohol consumption, 82.8% (*n* = 745) totally disagreed and 9.4% (*n* = 85) partially disagreed with “my alcohol consumption is excessive”, however, only 72.1% (*n* = 649) presented a moderate alcohol consumption. Smoking, illicit drug use, sleep habits, and stress did not seem to be overestimated, although their prevalence indicates difficulties in fulfilling beliefs, except for the use of illicit drugs.

There does not seem to be much previous similar literature that allows an adequate comparison of our results. Most of the studies carried out on patients’ perspectives on lifestyle behaviors embrace a qualitative design [56,57]. In these studies, patients recognize very important lifestyle behaviors as an integral part of self-care, but find it very difficult to integrate the necessary changes, although, a European cross-panel study on factors that influence the self-reporting of physical and cognitive health status with non-institutionalized adults aged 50 or over, in which Portugal participated with some data, suggests that comparisons of self-reported health between countries and age groups are subject to significant biases, while comparisons between genders are reliable for most European countries [58].

Similar to our findings, although with different methodologies, other studies had shown that fewer than 50% of primary care physicians consistently deliver specific guidance on nutrition, physical activity, or weight control [59]. In a survey of family doctors, 49% felt competent in prescribing weight loss programs, and only 14% believed that they were usually successful in helping obese patients to lose weight [60]. The EUROPREVIEW patient study reported that the discussion of healthy lifestyles did not occur according to risky drinkers’ points of view in approximately 40 to 60% of primary healthcare consultations. These patients considered that the family doctor initiated a discussion on alcohol less often (42.3%) than on smoking (63.4%), eating habits (59.2%), or physical activity (54.6%) [61]. Although primary care physicians recognize that it is necessary to recommend preventive and health promotion activities in practice, they do not perform as anticipated [62]. In this multi-center European study, the authors presented some possible barriers to the implementation of preventive and health promotion measures, with which we agree. The work overload, lack of time, lack of reimbursement, and the doctor’s own lifestyle can justify the reality found.

Although the results cannot be numerically comparable, integrating the point of view of health professionals [60] with the point of view of patients [61] seems very important. They are two distinct parts and, at the same time, they are very connected and related in the approach of lifestyle behaviors. The views of professionals and patients, both in primary healthcare, seem complementary, and the conclusions overlap, for example, an American doctor survey referring to negative stereotypes and difficulties in the correct behavior approach (to obesity) and a cross-sectional survey conducted in 22 European countries, in which patients with unhealthy lifestyles (especially high-risk drinkers) do not perceive the need to change their behaviours.

Within our Portuguese cohort, regarding lifestyle interventions, differences were found relating to gender, age, marital status, education level, general health status, and number of problems in the last 12 months. On the subject, the evidence has shown that socioeconomic factors are determinant and fundamental, which seem to vary according to the reality in which they are addressed [63]. It was curious to note that a smaller number of health problems in the last 12 months was associated with a less healthy diet and the practice of non-regular physical activity. However, participants with a greater number of problems generally have greater access to health services (more consultations and more professionals in the management of their problems), which may explain this situation. In addition, those with fewer problems may, in some way, be more permissive with their diet because, at the present time, they will not yet have developed significant health problems.

The authors consider that the data in this study are important for decision makers and those responsible for medical education in Portugal. It seems essential that medical schools define strategies to bridge the formative gap in this area and, for this, include curricular units related to the approach to lifestyle behaviors and facilitators of change. The training offered must include not only the basics, but also offer to train doctors in specialties and post-specialties. From the point of view of the authors, in a reality in which training on the topic is globally insufficient, training responses are needed at various levels so that, simultaneously and at various levels, one can contribute to a common goal.

There is also a need to implement multi-level measures, with particular emphasis on structuring policies that promote the prevention and control of non-communicable diseases [2,64]. The barriers and factors that affect the implementation of lifestyle interventions by primary care professionals must be considered from several points of view: intrapersonal (experiences, self-concept, beliefs, attitudes, motivation, skills, and knowledge), interpersonal (practice manager, practice staff, patient, and specialists), institutional factors (tools, practice organization, primary care organization, and the biomedical model), community factors (cultural context, mass media, the pharmaceutical industry, university, and social resources), and public policy (the health system model) [65,66]. In the particular case of Portugal, the primary healthcare pay per performance system may reinforce the practice of monitored activities over others, such as lifestyle interventions. Policymakers, in dialogue with researchers and physicians, should develop and create conditions for the applicability of consultation programs exclusively to address lifestyle interventions. The establishment of partnerships with professionals from complementary areas, municipalities, and community services, among others, will be of enormous value for an approach effectively capable of bringing health gains, as well as effective and sustainable solutions to the population.

The strengths of this study are its nationwide concept, with the questionnaire being applied during face-to-face interviews and with a representative sample of the adult general Portuguese population from mainland Portugal (900 participants). It should be noted that face-to-face interviews were also a strength of this study, since GDPR legally limited the use of previous databases for telephone contacts [67]. Furthermore, the sample was selected by the NUTS II geographical region quota method considering the distribution of the variables gender, age, and area of residence. Finally, this study included all lifestyle interventions: diet, physical activity, alcohol consumption, tobacco use, illicit drugs, sleep habits, screen activities, stress, and being sedentary. As with all research, this study has some limitations, primarily the cross-sectional study design and only including mainland residents. Furthermore, to the best of the authors’ knowledge, there is no validated questionnaire about the lifestyle interventions studied, which led to the use of a non-validated questionnaire specifically designed for the study. Cronbach’s α of the personal behavior self-assessment was 0.549, which is a low value for internal consistency, but still acceptable. Although another questionnaire has been applied to the Portuguese population, some of the aspects addressed in this study were not validated in the existing questionnaire, and it was not suitable for the purposes of this study [39]. To minimize recall bias, some of the questions on the questionnaire defined a short recall period. Self-reported data, some of which addressed sensitive issues, such as drug and alcohol consumption, could bias the results. Self-reported questionnaires are subjective, and recall bias must be considered when participants provide responses that depend on their ability to recall past events.

## 5. Conclusions

The patients’ personal beliefs about lifestyle in the prevention and treatment of some diseases in Portugal seem adequate. However, participants overestimate their self-assessment of personal behavior in their lifestyle, making difficulties suspected. Family doctors are probably overlooking these topics in their clinical practice, and do not ask/advise on lifestyle interventions, even to the extent that patients would like. It may be a priority that family doctors better clarify lifestyle recommendations, explain concepts, and empower and involve patients in the decision-making process about lifestyle interventions. The differences regarding gender, age, marital status, education level, general health status, and the number of problems in the last 12 months on lifestyle can be an excellent starting point for more complete knowledge in this area.

From a practical point of view, this study presents several suggestions and possibilities to improve the reality of patients and health professionals regarding lifestyle interventions: pilot projects in medical schools (including training content on a lifestyle approach), as well as strengthening postgraduate training, especially in the field of family medicine; inclusion of performance indicators with a focus on lifestyle interventions; development of consultation programs to address lifestyle interventions; and starting points for future investigations.

Future research directions should focus on the politics, economics, and policy aspects that may have an impact in this area, as considered in other studies [68], and which, in the specific case of Portugal, are related to the suggestions mentioned by the authors in this discussion. It will also be important to understand more broadly the relationships between lifestyle behaviors and clinical, physical, and sociodemographic variables.

## Figures and Tables

**Table 1:** Characterization of the sample (*n* = 900).

	*n* = 900
Nationality, *n* (%)	
Portuguese	859 (95.4)
Other nationality	41 (4.6)
Age (years), $\bar{x}\pm \mathrm{sd}$, Med, min-max	51.8 ± 18.1, 51, 20–99
Age groups (years), *n* (%)	
[20; 24]	58 (6.4)
[25; 29]	59 (6.6)
[30; 34]	63 (7.0)
[35; 39]	76 (8.4)
[40; 44]	89 (9.9)
[45; 49]	83 (9.2)
[50; 54]	81 (9.0)
[55; 59]	78 (8.7)
[60; 64]	72 (8.0)
[65; 69]	67 (7.4)
[70; 74]	57 (6.3)
≥75	117 (13.0)
Gender, *n* (%)	
Female	483 (53.7)
Male	417 (46.3)
Marital status, *n* (%)	
Single	187 (20.8)
Married	506 (56.2)
Married but legally separated	16 (1.8)
Divorced	85 (9.4)
Widowed	106 (11.8)
Highest level of education completed, *n* (%)	
None	24 (2.7)
Primary, first cycle	204 (22.7)
Primary, second cycle	76 (8.4)
Primary, third cycle	183 (20.3)
Secondary education	298 (33.1)
Higher education, bachelor	12 (1.3)
Higher education, graduation	78 (8.7)
Higher education, postgraduate studies	8 (0.9)
Higher education, masters	14 (1.6)
Higher education, PhD	3 (0.3)
Main occupation, *n* (%)	
Works on its own	146 (16.2)
Employed by others	424 (47.1)
Student	23 (2.6)
Doing military service	0 (0)
Homemaker	11 (1.2)
Retired	238 (26.4)
Unemployed	58 (6.4)
Professional sector (*n* = 570), *n* (%)	
Primary sector	3 (0.5)
Secondary sector	80 (14.0)
Tertiary sector	486 (85.3)
Health care beneficiary (multi response), *n* (%)	
SNS (National Health Service)	842 (93.6)
Private Health Insurance	128 (14.2)
Semi-private Health Service Assistance	74 (8.3)
Geographic distribution (NUTS II), *n* (%)	
North	330 (36.7)
Center	209 (23.2)
Metropolitan area of Lisbon	254 (28.2)
Alentejo	67 (7.4)
Algarve	40 (4.4)

NUTS II: Nomenclature of territorial units for statistical purposes.

**Table 2:** Self-perceived health general status and health problems of the sample.

**Health General Status** **, *n* (%)**	
Very good	83 (9.2)
Good	413 (45.9)
Reasonable	325 (36.1)
Poor	71 (7.9)
Very poor	8 (0.9)
Health problems last 12 months, *n* (%)	
None	356 (39.6)
Osteoarticular/muscular pain	349 (38.8)
Hypertension	178 (19.8)
Anxiety	153 (17.0)
Hypercholesterolemia	145 (16.1)
Overweight	140 (15.6)
Diabetes	99 (11.0)
Heart problems	72 (8.0)
Depression	58 (6.4)
Gastritis or peptic ulcer disease	49 (5.4)
Asthma and/or COPD	40 (4.4)
Stroke	16 (1.8)
Obesity	19 (2.1)
Cancer	12 (1.3)
Number of health problems last 12 months, *n* (%)	
1	189 (21.0)
2	141 (39.7)
3	105 (29.6)
4	45 (12.7)
≥ 5	64 (18.0)

**Table 3:** Response distribution by the level of agreement with statements about lifestyle and lifestyle in the context of family medicine.

	1 Strongly Disagree *n* (%)	2 Partially Disagree *n* (%)	3 Indifferent *n* (%)	4 Partially Agree *n* (%)	5 Strongly Agree *n* (%)	Med [95% CI]
**Diet**						
A healthy diet can prevent and help control some diseases.	0 (0.0)	1 (0.1)	8 (0.9)	108 (12.0)	783 (87.0)	5 [5; 5]
My diet is healthy.	13 (1.4)	46 (5.1)	159 (17.7)	373 (41.4)	309 (34.3)	4 [4; 4]
It is important that my family doctor asks/advises me about my diet.	57 (6.3)	18 (2.0)	84 (9.3)	233 (25.9)	508 (56.4)	5 [5; 5]
Usually, my family doctor asks/advises me about healthy diet.	258 (28.7)	93 (10.3)	73 (8.1)	171 (19.0)	305 (33.9)	4 [3; 4]
**Physical activity**						
Regular physical activity can prevent and help control some diseases.	2 (0.2)	3 (0.3)	5 (0.6)	104 (11.6)	786 (87.3)	5 [5; 5]
My physical activity is regular.	271 (30.1)	168 (18.7)	94 (10.4)	149 (16.6)	218 (24.2)	3 [2; 3]
It is important that my family doctor asks/advises me about my physical activity.	91 (10.1)	21 (2.3)	131 (14.6)	207 (23.0)	450 (50.0)	4.5 [4; 5]
Usually, my family doctor asks/advises me about physical activity.	306 (34.0)	81 (9.0)	98 (10.9)	149 (16.6)	266 (29.6)	3 [3; 3]
**Alcohol consumption**						
Excessive alcohol consumption can cause and aggravate some diseases.	1 (0.1)	0 (0)	3 (0.3)	66 (7.3)	830 (92.2)	5 [5; 5]
My alcohol consumption is excessive.	745 (82.8)	85 (9.4)	53 (5.9)	15 (1.7)	2 (0.2)	1 [1; 1]
It is important that my family doctor asks/advises me about my alcohol consumption.	132 (14.7)	36 (4.0)	154 (17.1)	167 (18.6)	411 (45.7)	4 [4; 4]
Usually, my family doctor asks/advises me about alcohol consumption.	402 (44.7)	85 (9.4)	97 (10.8)	114 (12.7)	202 (22.4)	2 [2; 2]
**Tobacco use**						
Smoking can cause and aggravate some diseases.	1 (0.1)	0 (0)	1 (0.1)	53 (5.9)	845 (93.9)	5 [5; 5]
I am an active smoker.	653 (72.6)	27 (3.0)	9 (1.0)	54 (6.0)	157 (17.4)	1 [1; 1]
It is important that my family doctor asks/advises me about smoking.	144 (16.0)	27 (3.0)	139 (15.4)	170 (18.9)	420 (46.7)	4 [4; 4]
Usually, my family doctor asks/advises me about smoking.	396 (44.0)	69 (7.7)	100 (11.1)	105 (11.7)	230 (25.6)	2 [2; 3]
**Illicit drugs**						
Illicit drugs can cause and aggravate some diseases.	2 (0.2)	0 (0)	7 (0.8)	38 (4.2)	853 (94.8)	5 [5; 5]
I do not use illicit drugs.	6 (0.7)	4 (0.4)	2 (0.2)	18 (2.0)	870 (96.7)	5 [5; 5]
It is important that my family doctor asks/advises me about illicit drugs.	265 (29.4)	41 (4.6)	150 (16.7)	116 (12.9)	328 (36.4)	3 [3; 4]
Usually, my family doctor asks/advises me about illicit drugs.	603 (67.0)	55 (6.1)	106 (11.8)	42 (4.7)	94 (10.4)	1 [1; 1]
**Sleep habits**						
Good quality sleep can prevent and help control some diseases.	1 (0.1)	1 (0.1)	2 (0.2)	75 (8.3)	821 (91.2)	5 [5; 5]
I have a good quality of sleep.	99 (11.0)	106 (11.8)	131 (14.6)	220 (24.4)	344 (38.2)	4 [4; 4]
It is important that my family doctor asks/advises me about sleep habits.	53 (5.9)	23 (2.6)	106 (11.8)	210 (23.3)	508 (56.4)	5 [5; 5]
Usually, my family doctor asks/advises me about sleep habits.	263 (29.2)	106 (11.8)	84 (9.3)	156 (17.3)	291 (32.3)	3 [3; 4]
**Screen activities**						
Excessive screen activities can cause and aggravate some diseases.	0 (0)	5 (0.6)	40 (4.4)	139 (15.4)	716 (79.6)	5 [5; 5]
I have excessive screen activities.	388 (43.1)	159 (17.7)	136 (15.1)	132 (14.7)	85 (9.4)	2 [2; 2]
It is important that my family doctor asks/advises me about screen activities.	187 (20.8)	63 (7.0)	192 (21.3)	142 (15.8)	316 (35.1)	4 [3; 4]
Usually, my family doctor asks/advises me about screen activities.	518 (57.6)	93 (10.3)	95 (10.6)	67 (7.4)	127 (14.1)	1 [1; 1]
**Stress**						
A high level of stress can cause and aggravate some diseases.	0 (0)	0 (0)	11 (1.2)	68 (7.6)	821 (91.2)	5 [5; 5]
I have a high level of stress.	340 (37.8)	166 (18.4)	151 (16.8)	162 (18.0)	81 (9.0)	2 [2; 2]
It is important that my family doctor asks/advises me about my level of stress.	68 (7.6)	30 (3.3)	117 (13.0)	219 (24.3)	466 (51.8)	5 [4; 5]
Usually, my family doctor asks/advises me about managing stress.	309 (34.3)	90 (10.0)	92 (10.2)	161 (17.9)	248 (27.6)	3 [3; 3]
**Sedentarism**						
Sedentarism can cause and aggravate some diseases.	0 (0)	0 (0)	6 (0.7)	85 (9.4)	809 (89.9)	5 [5; 5]
I am sedentary.	423 (47.0)	171 (19.0)	111 (12.3)	136 (15.1)	59 (6.6)	2 [1; 2]
It is important that my family doctor asks/advises me about sedentarism.	68 (7.6)	30 (3.3)	119 (13.2)	221 (24.6)	462 (51.3)	5 [4; 5]
Usually, my family doctor asks/advises me about sedentarism.	312 (34.7)	90 (10.0)	96 (10.7)	161 (17.9)	241 (26.8)	3 [3; 3]

Med: median value. 95% CI: 95% confidence interval.

**Table 4:** General characterization of the participants’ lifestyle patterns.

Diet, *n* (%)	
Healthy diet	126 (14.0)
Additional information on the topic:	
Body mass index (*n* = 891), *n* (%)	
Underweight	13 (1.4)
Normal	401 (44.6)
Overweight	358 (40.2)
Obesity	119 (13.4)
Physical activity, *n* (%)	
Regular physical activity	260 (28.9)
Alcohol consumption, *n* (%)	
Moderate alcohol consumption	649 (72.1)
Tobacco use, *n* (%)	
Non-smokers	669 (74.3)
Illicit drugs, *n* (%)	
Do not consume illicit drugs	888 (98.7)
Sleep habits, *n* (%)	
Good quality sleep	493 (54.8)
Screen activities, *n* (%)	
Moderate screen activities	586 (64.6)
Stress, *n* (%)	
Moderate stress level	584 (64.9)
Sedentarism, *n* (%)	
No sedentarism	455 (50.6)

**Table 5:** Association between lifestyle behaviors and bio-demographic variables.

		Dimension 1	Dimension 2	Dimension 3	Dimension 4
**Diet**	**Gender**	0.159 ^a^	0.376 ^a^	0.233 ^a^	0.269 ^a^
	Female	5 [5; 5]	4 [4; 5]	5 [4; 5]	4 [1; 5]
	Male	5 [5; 5]	4 [3.5; 5]	5 [4; 5]	4 [1; 5]
	**Age**	−0.011; 0.731 ^b^	0.060; 0.073 ^b^	0.074; 0.026 ^b,^*	0.158; <0.001 ^b,^*
	**Marital status**	0.454 ^a^	0.086 ^a^	0.290 ^a^	0.030 ^a,^*
	Married	5 [5; 5]	4 [4; 5]	5 [4; 5]	4 [1; 5]
	Others	5 [5; 5]	4 [3; 5]	5 [4; 5]	3 [1; 5]
	**Education level**	0.059 ^a^	0.626 ^a^	0.664 ^a^	0.318 ^a^
	High school or less	5 [5; 5]	4 [4; 5]	5 [4; 5]	4 [1; 5]
	Universitary education	5 [5; 5]	4 [3; 5]	5 [4; 5]	4 [1; 5]
	**Health general status**	0.984 ^c^	0.025 ^c,^*	0.020 ^c,^*	0.091 ^c^
	Very Good	5 [5; 5]	4 [4; 5]	4 [4; 5]	2 [1; 4]
	Good	5 [5; 5]	4 [4; 5]	5 [4; 5]	4 [1; 5]
	Reasonable	5 [5; 5]	4 [4; 5]	5 [4; 5]	4 [1; 5]
	Poor	5 [5; 5]	4 [3; 4]	4 [3; 5]	4 [1; 5]
	Very poor	5 [5; 5]	4 [2.5; 4]	5 [4; 5]	4 [1; 4]
	**NHPL 12 months**	0.054; 0.104 ^b^	−0.211; <0.001 ^b,^*	−0.024; 0.466 ^b^	0.011; 0.734 ^b^
**Physical activity**	**Gender**	0.984 ^a^	0.123 ^a^	0.031 ^a,^*	0.216 ^a^
	Female	5 [5; 5]	2 [1; 4]	5 [4; 5]	3 [1; 5]
	Male	5 [5; 5]	3 [1; 4]	4 [3; 5]	3 [1; 5]
	**Age**	−0.105; 0.022 ^b,^*	−0.292; <0.001 ^b,^*	−0.066; 0.150 ^b^	0.039; 0.393 ^b^
	**Marital status**	0.868 ^a^	0.036 ^a,^*	0.458 ^a^	0.138 ^a^
	Married	5 [5; 5]	2 [1; 4]	4.5 [4; 5]	3.5 [1; 5]
	Others	5 [5; 5]	3 [1; 5]	4.5 [3; 5]	3 [1; 5]
	**Education level**	0.418 ^a^	<0.001 ^a,^*	0.065 ^a^	0.547 ^a^
	High school or less	5 [5; 5]	2 [1; 4]	4 [3; 5]	3 [1; 5]
	Universitary education	5 [5; 5]	4 [2; 5]	5 [3; 5]	3 [1; 5]
	**Health general status**	0.269 ^c^	<0.001 ^c,^*	0.017 ^c,^*	0.046 ^c,^*
	Very Good	5 [5; 5]	4 [2; 5]	4 [3; 5]	2 [1; 4]
	Good	5 [5; 5]	3 [2; 5]	5 [3; 5]	3 [1; 5]
	Reasonable	5 [5; 5]	2 [1; 4]	5 [4; 5]	4 [1; 5]
	Poor	5 [5; 5]	2 [1; 3]	4 [3; 5]	3 [1; 4]
	Very poor	5 [4.25; 5]	1 [1; 1.75]	4.5 [3.25; 5]	1.5 [1; 4]
	**NHPL 12 months**	0.027; 0.554 ^b^	−0.301; <0.001 ^b,^*	−0.116; 0.010 ^b,^*	−0.043; 0.342 ^b^
**Alcohol consumption**	**Gender**	0.510 ^a^	<0.001 ^a,^*	0.103 ^a^	0.003 ^a,^*
	Female	5 [5; 5]	5 [5; 5]	4 [3; 5]	2 [1; 4]
	Male	5 [5; 5]	5 [4; 5]	4 [3; 5]	3 [1; 5]
	**Age**	0.010; 0.757 ^b^	0.115; 0.001 ^b,^*	−0.048; 0.148 ^b^	0.039; 0.239 ^b^
	**Marital status**	0.173 ^a^	0.744 ^a^	0.218 ^a^	0.022 ^a,^*
	Married	5 [5; 5]	5 [5; 5]	4 [3; 5]	2 [1; 4]
	Others	5 [5; 5]	5 [5; 5]	4 [3; 5]	2 [1; 4]
	**Education level**	0.437 ^a^	0.357 ^a^	0.205 ^a^	0.084 ^a^
	High school or less	5 [5; 5]	5 [5; 5]	4 [3; 5]	2 [1; 4]
	Universitary education	5 [5; 5]	5 [5; 5]	5 [3; 5]	1 [1; 4]
	**Health general status**	0.874 ^c^	0.092 ^c^	0.005 ^c,^*	0.016 ^c,^*
	Very Good	5 [5; 5]	5 [5; 5]	4 [3; 5]	1 [1; 4]
	Good	5 [5; 5]	5 [5; 5]	4 [3; 5]	2 [1; 4]
	Reasonable	5 [5; 5]	5 [5; 5]	5 [3; 5]	2 [1; 4]
	Poor	5 [5; 5]	5 [5; 5]	3 [1; 5]	1 [1; 3]
	Very poor	5 [5; 5]	5 [5; 5]	5 [3; 5]	1 [1; 2.75]
	**NHPL 12 months**	0.073; 0.029 ^b,^*	0.109; 0.001 ^b,^*	−0.175; <0.001 ^b,^*	−0.142; <0.001 ^b,^*
**Tobacco use**	**Gender**	0.327 ^a^	<0.001 ^a,^*	0.170 ^a^	0.001 ^a,^*
	Female	5 [5; 5]	5 [5; 5]	4 [3; 5]	2 [1; 4]
	Male	5 [5; 5]	5 [2; 5]	4 [3; 5]	3 [1; 5]
	**Age**	0.012; 0.721 ^b^	0.226; <0.001 ^b,^*	−0.151; <0.001 ^b,^*	−0.118; <0.001 ^b,^*
	**Marital status**	0.264 ^a^	0.003 ^a,^*	0.046 ^a,^*	0.199 ^a^
	Married	5 [5; 5]	5 [5; 5]	4 [3; 5]	2 [1; 5]
	Others	5 [5; 5]	5 [2; 5]	4 [3; 5]	2 [1; 4]
	**Education level**	0.210 ^a^	0.095 ^a^	0.329 ^a^	0.372 ^a^
	High school or less	5 [5; 5]	5 [3; 5]	4 [3; 5]	2 [1; 5]
	Universitary education	5 [5; 5]	5 [5; 5]	5 [3; 5]	1 [1; 5]
	**Health general status**	0.354 ^c^	<0.001 ^c,^*	0.004 ^c,^*	0.014 ^c,^*
	Very Good	5 [5; 5]	5 [5; 5]	4 [3; 5]	1 [1; 4]
	Good	5 [5; 5]	5 [2; 5]	4 [3; 5]	3 [1; 5]
	Reasonable	5 [5; 5]	5 [5; 5]	4 [3; 5]	2 [1; 4]
	Poor	5 [5; 5]	5 [5; 5]	3 [1; 5]	1 [1; 3]
	Very poor	5 [5; 5]	5 [5; 5]	4.5 [1.5; 5]	1 [1; 2.5]
	**NHPL 12 months**	0.103; 0.002 ^b,^*	0.090; 0.007 ^b,^*	−0.237; <0.001 ^b,^*	−0.203; <0.001 ^b,^*
**Ilicit drugs**	**Gender**	0.188 ^a^	0.002 ^a,^*	0.895 ^a^	0.297 ^a^
	Female	5 [5; 5]	5 [5; 5]	3 [1; 5]	1 [1; 3]
	Male	5 [5; 5]	5 [5; 5]	3 [1; 5]	1 [1; 3]
	**Age**	0.002; 0.955 ^b^	0.042; 0.206 ^b^	−0.162; <0.001 ^b,^*	−0.099; 0.003 ^b,^*
	**Marital status**	0.278 ^a^	0.065 ^a^	0.591 ^a^	0.602 ^a^
	Married	5 [5; 5]	5 [5; 5]	3 [1; 5]	1 [1; 3]
	Others	5 [5; 5]	5 [5; 5]	3 [1; 5]	1 [1; 3]
	**Education level**	0.998 ^a^	0.650 ^a^	0.045 ^a,^*	0.947 ^a^
	High school or less	5 [5; 5]	5 [5; 5]	3 [1; 5]	1 [1; 3]
	Universitary education	5 [5; 5]	5 [5; 5]	4 [2; 5]	1 [1; 3]
	**Health general status**	0.070 ^c^	0.716 ^c^	0.072 ^c^	0.349 ^c^
	Very Good	5 [5; 5]	5 [5; 5]	4 [3; 5]	1 [1; 3]
	Good	5 [5; 5]	5 [5; 5]	3 [1; 5]	1 [1; 3]
	Reasonable	5 [5; 5]	5 [5; 5]	4 [1; 5]	1 [1; 3]
	Poor	5 [5; 5]	5 [5; 5]	3 [1; 4]	1 [1; 2]
	Very poor	5 [5; 5]	5 [5; 5]	2.5 [1; 5]	1 [1; 1]
	**NHPL 12 months**	0.090; 0.007 ^b,^*	0.068; 0.041 ^b,^*	−0.095; 0.004 ^b,^*	−0.202; <0.001 ^b,^*
**Sleep habits**	**Gender**	0.423 ^a^	0.005 ^a,^*	0.006 ^a,^*	0.036 ^a,^*
	Female	5 [5; 5]	4 [2; 5]	5 [4; 5]	4 [1; 5]
	Male	5 [5; 5]	4 [3; 5]	5 [4; 5]	3 [1; 5]
	**Age**	−0.031; 0.346 ^b^	−0.168; <0.001 ^b,^*	0.136; <0.001 ^b,^*	0.215; <0.001 ^b,^*
	**Marital status**	0.417 ^a^	0.280 ^a^	0.895 ^a^	0.644 ^a^
	Married	5 [5; 5]	4 [3; 5]	5 [4; 5]	3.5 [1; 5]
	Others	5 [5; 5]	4 [2.75; 5]	5 [4; 5]	3 [1; 5]
	**Education level**	0.989 ^a^	0.005 ^a,^*	0.567 ^a^	0.019 ^a,^*
	High school or less	5 [5; 5]	4 [3; 5]	5 [4; 5]	4 [1; 5]
	Universitary education	5 [5; 5]	5 [3; 5]	5 [3; 5]	3 [1; 4]
	**Health general status**	0.948 ^c^	<0.001 ^c,^*	<0.001 ^c,^*	<0.001 ^c,^*
	Very Good	5 [5; 5]	5 [4; 5]	4 [3; 5]	2 [1; 4]
	Good	5 [5; 5]	4 [3; 5]	5 [4; 5]	3 [1; 5]
	Reasonable	5 [5; 5]	4 [2; 5]	5 [4; 5]	4 [2; 5]
	Poor	5 [5; 5]	3 [2; 4]	4 [3; 5]	4 [1; 5]
	Very poor	5 [5; 5]	1.5 [1; 3.75]	5 [4.25; 5]	3 [1; 4.75]
	**NHPL 12 months**	0.008; 0.817 ^b^	−0.420; <0.001 ^b,^*	0.017; 0.602 ^b^	0.026; 0.433 ^b^
**Screen activities**	**Gender**	0.030 ^a,^*	0.663 ^a^	0.756 ^a^	0.632 ^a^
	Female	5 [5; 5]	4 [3; 5]	4 [2; 5]	1 [1; 3]
	Male	5 [5; 5]	4 [3; 5]	4 [2; 5]	1 [1; 3]
	**Age**	−0.033; 0.320 ^b^	0.220; <0.001 ^b,^*	−0.066; 0.047 ^b,^*	−0.003; 0.924 ^b^
	**Marital status**	0.109 ^a^	0.123 ^a^	0.012 ^a,^*	0.044 ^a,^*
	Married	5 [5; 5]	4 [3; 5]	4 [2; 5]	1 [1; 3]
	Others	5 [5; 5]	4 [2; 5]	3 [2; 5]	1 [1; 3]
	**Education level**	0.341 ^a^	<0.001 ^a,^*	0.662 ^a^	0.056 ^a^
	High school or less	5 [5; 5]	4 [3; 5]	4 [2; 5]	1 [1; 3]
	Universitary education	5 [5; 5]	3 [2; 5]	4 [2; 5]	1 [1; 2]
	**Health general status**	0.040 ^c,^*	0.064 ^c^	<0.001 ^c,^*	0.006 ^c,^*
	Very Good	5 [4; 5]	4 [2; 5]	3 [1; 4]	1 [1; 2]
	Good	5 [5; 5]	4 [2; 5]	4 [2.5; 5]	1 [1; 3]
	Reasonable	5 [5; 5]	4 [3; 5]	4 [2; 5]	1 [1; 3]
	Poor	5 [4; 5]	4 [3; 5]	3 [1; 4]	1 [1; 3]
	Very poor	5 [5; 5]	5 [2.5; 5]	4 [2; 5]	1 [1; 1.75]
	**NHPL 12 months**	−0.024; 0.467 ^b^	0.009; 0.788 ^b^	−0.182; <0.001 ^b,^*	−0.192; <0.001 ^b,^*
Stress	**Gender**	0.723 ^a^	0.024 ^a,^*	0.417 ^a^	0.142 ^a^
	Female	5 [5; 5]	4 [2; 5]	5 [4; 5]	3 [1; 5]
	Male	5 [5; 5]	4 [3; 5]	5 [3; 5]	3 [1; 5]
	**Age**	−0.028; 0.408 ^b^	0.139; <0.001 ^b,^*	0.035; 0.288 ^b^	0.080; 0.016 ^b,^*
	**Marital status**	0.555 ^a^	0.399 ^a^	0.091 ^a^	0.088 ^a^
	Married	5 [5; 5]	4 [2; 5]	5 [4; 5]	3 [1; 5]
	Others	5 [5; 5]	4 [2; 5]	4 [3; 5]	3 [1; 5]
	**Education level**	0.939 ^a^	0.003 ^a,^*	0.634 ^a^	0.090 ^a^
	High school or less	5 [5; 5]	4 [2; 5]	5 [4; 5]	3 [1; 5]
	Universitary education	5 [5; 5]	3 [2; 5]	5 [3; 5]	2 [1; 4]
	**Health general status**	0.721 ^c^	0.086 ^c^	0.035 ^c,^*	0.030 ^c,^*
	Very Good	5 [5; 5]	4 [3; 5]	4 [3; 5]	2 [1; 4]
	Good	5 [5; 5]	4 [2; 5]	5 [4; 5]	3 [1; 5]
	Reasonable	5 [5; 5]	4 [2; 5]	5 [4; 5]	3 [1; 5]
	Poor	5 [5; 5]	3 [2; 5]	4 [4; 5]	4 [1; 5]
	Very poor	5 [5; 5]	3 [2; 3.75]	5 [4.25; 5]	4 [1; 4]
	**NHPL 12 months**	0.045; 0.181 ^b^	−0.173; <0.001 ^b,^*	−0.042; 0.214 ^b^	−0.043; 0.196 ^b^
**Sedentarism**	**Gender**	0.699 ^a^	0.289 ^a^	0.162 ^a^	0.962 ^a^
	Female	5 [5; 5]	4 [3; 5]	5 [4; 5]	3 [1; 5]
	Male	5 [5; 5]	4 [3; 5]	4 [3; 5]	3 [1; 5]
	**Age**	−0.030; 0.371 ^b^	−0.267; <0.001 ^b,^*	0.056; 0.095 ^b^	0.151; <0.001 ^b,^*
	**Marital status**	0.250 ^a^	0.065 ^a^	0.093 ^a^	0.749 ^a^
	Married	5 [5; 5]	4 [3; 5]	5 [4; 5]	3 [1; 5]
	Others	5 [5; 5]	4 [3; 5]	4 [3; 5]	3 [1; 5]
	**Education level**	0.817 ^a^	0.664 ^a^	0.480 ^a^	0.331 ^a^
	High school or less	5 [5; 5]	4 [3; 5]	5 [4; 5]	3 [1; 5]
	Universitary education	5 [5; 5]	4 [3; 5]	5 [3; 5]	2 [1; 5]
	**Health general status**	0.446 ^c^	<0.001 ^c,^*	0.025 ^c,^*	<0.001 ^c,^*
	Very Good	5 [5; 5]	5 [4; 5]	4 [3; 5]	1 [1; 3]
	Good	5 [5; 5]	5 [3; 5]	5 [4; 5]	3 [1; 5]
	Reasonable	5 [5; 5]	4 [2; 5]	5 [4; 5]	3 [1; 5]
	Poor	5 [5; 5]	3 [2; 5]	4 [3; 5]	4 [1; 4]
	Very poor	5 [5; 5]	2 [2; 4.75]	5 [4.25; 5]	3.5 [1; 4.75]
	**NHPL 12 months**	0.064; 0.055 ^b^	−0.251; <0.001 ^b,^*	−0.043; 0.202 ^b^	0.005; 0.870 ^b^

a: Mann–Whitney test. b: Spearman coefficient and the respective *p*-value. c: Kruskal–Wallis test. * Significant at 5%. NHPL 12 months: number of health problems in the last 12 months. Dimension 1: personal belief about each lifestyle intervention (can prevent and help control vs. can cause and aggravate some diseases, as appropriate); dimension 2: personal behavior self-assessment (if, in the personal case, each lifestyle intervention is healthy or not); dimension 3: level of importance given to the family doctor to address each lifestyle intervention; and dimension 4: if, in the personal case, something was usual or not, the family doctor asks/advises on each lifestyle intervention.

## Data Availability

Complete de-identified participants’ data sets will be available with the publication if a request is sent to rosaliapascoa@hotmail.com. The data will be made available to anyone requesting it, after indicating the reason for the need for access and after the approval of all authors.

## Supplementary Materials

The following are available online at https://www.mdpi.com/1660-4601/18/6/2981/s1, File S1. The questionnaire used for data collection; Table S1. Characterization of the pre-test participants; Table S2. Sociodemographic data of the Portuguese population, obtained from the PORDATA website, www.pordata.pt; Table S3. Comparison between levels of agreement of personal beliefs and personal self-report realities for each lifestyle intervention; and, Table S4. Characterization of lifestyle patterns of participants.
