# Supplementary material for: Patients’ Perspectives about Lifestyle Behaviors and Health in the Context of Family Medicine: A Cross-Sectional Study in Portugal
Source: Int J Environ Res Public Health. 2021 Mar 14;18(6):2981. doi: 10.3390/ijerph18062981 (PMC8001049; doi:10.3390/ijerph18062981)
Supplement: Supplementary file 1 [file ijerph-18-02981-s001.zip › Suplementary files_13.03.2021/File S1_Questionnaire.pdf]

Faculty of Medicine, University of Porto

Questionnaire number |__________|

Introductory note

Dear Sir/Madam,

We would like to invite you to answer a questionnaire as part of a research project of the Faculty of Medicine of the University of Porto. The main goal is to understand your opinion about lifestyle.

The questionnaire is anonymous, meaning that your identity will never be recorded or revealed. It consists of 3 parts: the first on general health; the second on lifestyle and the third on sociodemographic data. The expected duration of the questionnaire is 20 to 25 minutes.

Your collaboration is voluntary. At any time, if you wish, you may give up answering the questions without any repercussions. Still, we would like to tell you that your collaboration is of utmost importance to the success of this study.

Want some further clarification?

Will you willingly answer this questionnaire?

Part I – General state of health

The following two questions ask your opinion about your health. Pay attention to each question and answer as honestly as possible. **Most important is your opinion.**

1. **Overall, how do you consider your health?**

   ① Very good ② Good ③ Reasonable ④ Bad ⑤ Very bad

2. **Indicate if during the last 12 months you have had any of the following health problems** (multi response)**:**

1	High blood pressure (hypertension)
2	Stroke
3	High blood cholesterol
4	Diabetes
5	Heart problems (infarction, angina pectoris, heart valve disease, heart failure)
6	Back, bone, joint or muscle pain
7	Chronic bronchitis, asthma, chronic obstructive pulmonary disease or emphysema
8	Gastritis or stomach ulcer/duodenum
9	Anxiety
10	Depression
11	Overweight
12	Obesity
13	Cancer
14	None of these

Part II – Lifestyle

In this part, we want to know what you think about lifestyle. When answering, do not worry about what is correct or not. Just answer according to what your personal opinion dictates.

Please indicate your agreement with each of the following sentences. Rate on a scale of 1 to 5, corresponding **1 to "Strongly disagree" and 5 to "Strongly agree"**.

		Strongly disagree	Partially disagree	Indifferent	Partially agree	Strongly agree
**Diet**						
1.	A healthy diet can prevent and help control some diseases.	1	2	3	4	5
2.	My diet is healthy.	1	2	3	4	5
3.	It is important that my family doctor asks/advises me about my diet.	1	2	3	4	5
4.	Usually, my family doctor asks/advises me about a healthy diet.	1	2	3	4	5
**Physical activity**						
1.	Regular physical activity can prevent and help control some diseases.	1	2	3	4	5
2.	My physical activity is regular.	1	2	3	4	5
3.	It is important that my family doctor asks/advises me about my physical activity.	1	2	3	4	5
4.	Usually, my family doctor asks/advises me about physical activity.	1	2	3	4	5
**Alcohol intake**						
1.	Excessive alcohol intake can cause and aggravate some diseases.	1	2	3	4	5
2.	My alcohol intake is excessive.	1	2	3	4	5
3.	It is important that my family doctor asks/advises me about my alcohol intake.	1	2	3	4	5
4.	Usually, my family doctor asks/advises me about alcohol intake.	1	2	3	4	5
**Tobacco use**						
1.	Smoking can cause and aggravate some diseases.	1	2	3	4	5
2.	I am an active smoker.	1	2	3	4	5
3.	It is important that my family doctor asks/advises me about smoking.	1	2	3	4	5
4.	Usually, my family doctor asks/advises me about smoking.	1	2	3	4	5
**Illicit drugs**						
1.	Illicit drugs can cause and aggravate some diseases.	1	2	3	4	5
2.	I do not use illicit drugs.	1	2	3	4	5
3.	It is important that my family doctor asks/advises me about illicit drugs.	1	2	3	4	5
4.	Usually, my family doctor asks/advises me about illicit drugs.	1	2	3	4	5
**Sleep habits**						
1.	Good quality sleep can prevent and help control some diseases.	1	2	3	4	5
2.	I have a good quality of sleep.	1	2	3	4	5
3.	It is important that my family doctor asks/advises me about sleep habits.	1	2	3	4	5
4.	Usually, my family doctor asks/advises me about sleep habits.	1	2	3	4	5
**Screen activities (watching television; using the computer, mobile phone, game consoles, …)**						
1.	Excessive screen activities can cause and aggravate some diseases.	1	2	3	4	5
2.	I have excessive screen activities.	1	2	3	4	5
3.	It is important that my family doctor asks/advises me about screen activities.	1	2	3	4	5
4.	Usually, my family doctor asks/advises me about screen activities.	1	2	3	4	5
**Stress**						
1.	A high level of stress can cause and aggravate some diseases.	1	2	3	4	5
2.	I have a high level of stress.	1	2	3	4	5
3.	It is important that my family doctor asks/advises me about my level of stress.	1	2	3	4	5
4.	Usually, my family doctor asks/advises me about managing stress.	1	2	3	4	5
**Sedentarism (amount of time without physical activity)**						
1.	Sedentarism can cause and aggravate some diseases.	1	2	3	4	5
2.	I am sedentary.	1	2	3	4	5
3.	It is important that my family doctor asks/advises me about sedentarism.	1	2	3	4	5
4.	Usually, my family doctor asks/advises me about sedentarism.	1	2	3	4	5

1. **Please indicate:**

1.1. **Weight** (without clothes and without shoes): ____ (Kg)

1.2. **Height** (without shoes):____ (cm)

2. **How many meals do you usually eat per day? Consider breakfast, lunch, and dinner as main meals and snacks and supper as intermediate meals.**

Number of main meals: ____

Number os intermediate meals: ____

2.1. In the main meals you had yesterday, you consumed (multi response)**:**

1	Milk, yogurt or cheese
2	Vegetable soup
3	Bread
4	Beef
5	Fish
6	Potatoes, rice or pasta
7	Beans or chickpeas
8	Cakes, cookies, chocolates or desserts
9	Soft drinks, with or without gas
10	Natural juices, made from fresh fruit
11	Vegetables (salads, cooked vegetables)
12	Fast food (meal prepared and served quickly following a standardized and massified method, whether or not it can be eaten at the place of purchase)
13	Pre-cooked / industrially prepared meal (meal prepared according to an industrialized method that includes cooking partially or completely the same, keeping it according to refrigeration, freezing, vacuuming or canning processes, and before being consumed requires the completion of cooking heat treatment such as oven, microwave or short-term frying.)
14	None of these

2.2. How often do you eat fruit, including juices made from fresh fruit but excluding juices made from concentrates? (Do not consider canned or dried fruits.)

① Once or more a day
② 4 to 6 times a week
③ 1 to 3 times a week
④ 1 to 3 times a week
⑤ Never → **Go to question 2.3.**

2.2.1. Usually, how many servings of fruit do you consume per day?

Number of servings of fruit:____

2.3. How often, do you eat vegetables or salads, excluding potatoes and juices made from concentrates? (Also consider the juices made from fresh vegetables, canned vegetables, legumes (beans, lentils), soups (heat or cold) and vegetarian dishes.)

① Once or more a day
② 4 to 6 times a week
③ 1 to 3 times a week
④ 1 to 3 times a week
⑤ Never → **Go to question 3.**

2.3.1. Usually, how many servings of vegetables or salads do you consume per day?

Number of servings of vegetables anda salads:____

3. Think about the time you spend on activities such as working, caring for the house, caring for family members, studying, volunteering, and so on. Do not consider leisure or sports activities.
From the following options select the one that best describes the main way you perform the activities mentioned. (If you do several activities, consider the one that you spend the most time with.)

① Sitting or standing in activities involving slight physical exertion
② On the move or on tasks that require moderate physical exertion
③ In heavy or physically demanding jobs
④ Do not perform any of the indicated activities

3.1. In a normal week during your travels, how many days are you walking for at least 10 minutes straight? Also, consider the weekend. (If you do not walk or do so for less than 10 minutes straight, record 0.)

Number of days:____ → **(If you answered 0, go to question 3.2.)**

3.1.1. And in a normal, in your travels, how long are you walking? (If your travel time varies greatly throughout the week, please state the average time per day.

① Less than 10 minutes
② 10 to 29 minutes
③ 30 to 59 minutes
④ 1 hour to less than 2 hours
⑤ 2 hours to less than 3 hours
⑥ 3 hours or more

3.2. Now think of sports or leisure activities. In a normal week, how many days does physical activity last for less than 10 minutes in a row? Consider the weekend. (Examples: walking, playing ball, jogging, biking, or swimming. If you do not exercise or do less than 10 minutes in a row, record 0.)

Number of days:____ → **(If you answered 0, go to question 4.)**

3.2.1. On this (these) day(s), on average, how long do you practice physical activity?

① 10 to 29 minutes
② 30 to 59 minutes
③ 1 hour to less than 2 hours
④ 2 horas to less than 3 hours
⑤ 3 hours or more

3.3. In a normal week, how many days do you practice muscle-building activities, such as resistance training or bodybuilding? (If you do not engage in muscle-building activities, record 0. Consider all muscle-building activities, even if they have been considered before.)

Number of days:____

4. In the last 12 months, how often have you consumed alcoholic beverages of any kind (beer, wine, spirits, cocktails, liquors, mixtures of alcoholic beverages, among others)?

① Everyday or almost every day (Choose this option if you drank in the 365 days of the last 12 months or usually more than 6 days a week.)
② 5 to 6 days a week
③ 3 to 4 days a week
④ 1 to 2 days a week
⑤ 2 to 3 days a month → **(Go to question 4.2.)**
⑥ Once a month → **(Go to question 4.2.)**
⑦ Less than once a month → **(Go to question 4.2.)**
⑧ You have not consumed in the last 12 months because you have stopped drinking (**Choose this option if you have not consumed alcohol in the last 12 months, but have had at least one drink throughout your life without just proving it**.) → **(Go to question 5.)**
⑨ Never consumed, or only occasionally to taste (**Choose this option if you have never consumed a drink in your life, or, at most, sipped occasionally for the purpose of tasting the drink**.) → **(Go to question 5.)**

4.1. Usually, between Monday and Thursday, how many days do you drink alcohol?

① 4 days
② 3 days

③ 2 days ④ 1 day
⑤ On neither day → **(Go to question 4.2.)**

4.1.1. And on those days, on average, how many alcoholic drinks do you consume?

① 16 or more drinks per day ② 10 to 15 drinks per day
③ 6 to 9 drinks per day ④ 4 to 5 drinks per day
⑤ 3 drinks per day ⑥ 2 drinks per day
⑦ 1 drink per day

4.2. And usually, between Friday and Sunday, how many days do you drink alcohol?

① 3 days ② 2 days
③ 1 day ④ On neither day → **(Go to question 4.3.)**

4.2.1. And on one of those days, on average, how many alcoholic drinks do you consume?

① 16 or more drinks per day ② 10 to 15 drinks per day
③ 6 to 9 drinks per day ④ 4 to 5 drinks per day
⑤ 3 drinks per day ⑥ 2 drinks per day
⑦ 1 drink per day

4.3. In the last 12 months, have you consumed 6 or more alcoholic beverages on a single occasion or event? For example, at a party, at a meal, going out with friends or alone at home. (Consider any type of beverage: beer, wine, spirits, cocktails, liqueurs, alcohol mixes, home-made alcoholic beverages, etc.)

① Every day or almost every day ② 5 to 6 days per week
③ 3 to 4 days per week ④ 1 to 2 days per week
⑤ 2 to 3 days per month ⑥ Once a month
⑦ Less than once a month ⑧ Not in the last 12 months
⑨ Never in life

5. Do you smoke? (Consider any kind of tobacco, except the electronic cigarette. Answer regardless of regular consumption)
① Yes
② No → **5.0.1. Have you ever smoked? (Consider any kind of tobacco, except the electronic cigarette. Answer regardless of regular consumption)**

① No → **(Go to question 5.4.)**
② Yes → **5.0.2. How long have you quit smoking?**
① Less than 1 year → **(Go to question 5.4.)**
② More than 1 year → **(Go to question 5.4.)**

5.1. You smoke:
① Daily → **5.1.1. At what age did you start to smoke daily?** Age:____
② Occasionally

5.2. On average how many cigarettes do you smoke per day? (Consider any kind of tobacco, except the electronic cigarette. Answer regardless of regular consumption)

Number of cigarettes: __

5.3. Do you use an electronic cigarette? (Electronic mechanical device that produces inhalable vapor, with or without nicotine, also known as e-cigarette.)

① Yes ② No

5.4. How often are you indoors where other people smoke? (Consider only the smoke produced by other people. Examples of enclosed spaces: home, work, public spaces, restaurants, car, etc.)

① Daily
② Occasionally
③ Never → **(Go to question 6.)**

5.5. And usually, how long are you exposed?
① Less than 1 hour per day
② 1 hour or more

5.6. And this exposure to indoor tobacco smoke happens mostly where?

① At home ② In the car
③ At work ④ In leisure places
⑤ Other locations

6. Do you use illicit drugs? (Consider any type of drug: cannabis, cocaine (crack, ..), heroin, amphetamines (ecstasy, ..), hallucinogens, among others,… Answer regardless of regular use.)

① Yes
② No → **6.0.1. Have you ever consumed? (Answer regardless of regular use.)**
① No → **(Go to question 7.)**
② Yes → **6.0.2. How long have you stopped consuming?**
① Less than 1 year → **(Go to question 7.)**
② 1 year or more → **(Go to question 7.)**

6.1. You consume:
① Daily → **6.1.1. At what age did you start to smoke daily?** Age: ____
② Occasionally

6.2. On average how many consumptions do you make per week? (Consider any type of drug. Answer regardless of regular use.)

Number of consumptions: ____

7. In the last 2 weeks, how often have you had sleep problems (difficulty falling asleep, little sleep or too much sleep)?

① Every day
② Most part of the days
③ Some days
④ A few days
⑤ Never

7.1. In the last 2 weeks, how often have you needed to take sleeping medication?

① Every day
② Most part of the days
③ Some days
④ A few days
⑤ Never

7.2. In the last 2 weeks, how often have you got restful sleep?
① Every day
② Most part of the days
③ Some days
④ A few days
⑤ Never

8. In a normal week, how many days do you perform screen activities (watching TV; using a computer, mobile phone, game consoles, etc.)? Also, consider the weekend.

Number of days: ____→ **(If you answered 0, go to question 9.)**

8.1. And on a normal day, how much time do you use for screen activities? (If you do more than one activity, consider the one you do most often and tick only one answer. If your usage time varies a lot over the week, indicate the average time per day.)

① Less than 10 minutes
② 10 to 29 minutes
③ 30 to 59 minutes
④ 1 hour to less than 2 hours
⑤ 2 hours to less than 3 hours
⑥ 3 hours or more

8.2. On which device do you usually do that screen activities? (If you use more than one type of device, consider what you use most often and tick only one answer.)

① Television ②Computer
③ Mobile phone ④Game consoles

8.3. In the last 2 weeks, how often did you do less than you wanted in your work or daily activities because you were doing screen activities?

① Every day
② Most part of the days
③ Some days
④ A few days
⑤ Never

9. In the last 2 weeks, how often have you done less than you wanted in your work or daily activities because you felt anxious/nervous?

① Every day
② Most part of the days
③ Some days
④ A few days
⑤ Never

9.1. In the last 2 weeks, how often have you felt calm and peaceful?

① Every day
② Most part of the days
③ Some days
④ A few days
⑤ Never

9.2. In the last 2 weeks, how often did you feel anxious/nervous?

① Every day
② Most part of the days
③ Some days
④ A few days
⑤ Never

9.3. In the last 2 weeks, have you had to take medication to control anxiety/nervousness?

① Every day
② Most part of the days
③ Some days
④ A few days
⑤ Never

Part III – Socio-demographic data

This part of the questionnaire aims to collect some data related to you, your education and your professional activity.

1. **What is your nationality?**
   ① Portuguese
   ② Foreign. Which one:______________
   ③ Stateless (without nationality)
   ④ Does not know

2. **How old are you?** ______________ years

3. **Your gender:**
   ① Female ② Male

4. **What is your marital status?**
   ① Single
   ② Married
   ③ Married but legally separated
   ④ Divorced
   ⑤ Widowed
   ⑥ Does not know

5. **What is the highest level of education you have completed?**
   ① None
   ② Primary, 1st cycle (4th year complete)
   ③ Primary, 2nd cycle (6th year complete)
   ④ Primary, 3rd cycle (9th year complete)
   ⑤ Secondary (12th complete)
   ⑥Higher education, bachelor
   ⑦ Higher education, graduation
   ⑧ Higher education, postgraduate studies
   ⑨ Higher education, masters
   ⑩ Higher education, PhD
   ⑪ Does not know

6. Of the following categories, which best describes your main occupation?

① Works on it owns
② Works for others
③ Student
④ Doing military service
⑤ Homemaker
⑥ Retired
⑦ Unemployed
⑧ Does not know

7. What is your main profession?

Indicate:

____

① Does not have a profession
② Does not Know

8. Which of the following is a health care recipient?

① ADSE (State Health Service Assistance)
② SSMJ (Justice Ministry Service)
③ IASFA (Institute of Social Action of the Armed Forces)
④ SAD/PSP (PSP Disease Assistance Services)
⑤ SAD/GNR (GNR Disease Assistance Services)
⑥ SAMS (Banking Operations Department)
⑦ Private Health Insurance
⑧ SNS (National Health System)
⑨ Others (indicate): ______________________
⑩ Does not Know

9. What is your municipality of residence?

Indicate:

____

Thank you so much for your cooperation!
