# Supplementary material for: Patients’ Perspectives about Lifestyle Behaviors and Health in the Context of Family Medicine: A Cross-Sectional Study in Portugal
Source: Int J Environ Res Public Health. 2021 Mar 14;18(6):2981. doi: 10.3390/ijerph18062981 (PMC8001049; doi:10.3390/ijerph18062981)
Supplement: Supplementary file 1 [file ijerph-18-02981-s001.zip › Suplementary files_13.03.2021/Table S1_Characterization of the pre-test participants.docx]

**Table S1**. Characterization of the pre-test participants (n = 20).

|  | **n = 20** |
| --- | --- |
| **Nationality**, n (%) |  |
| Portuguese | 20 (100) |
| Foreign | 0 (0) |
| **Age** (years), $\bar{x}\pm s$ $\bar{x}\pm sd$, Med, min-max | 47.1±17.1, 45, 23-85 |
| **Gender**, n (%) |  |
| Female | 11 (55) |
| Male | 9 (45) |
| **Marital status**, n (%) |  |
| Single | 2 (10) |
| Married | 14 (70) |
| Married but legally separated | 1 (5) |
| Divorced | 3 (15) |
| Widowed | 0 (0) |
| **Highest level of education completed**, n (%) |  |
| None | 0 (0) |
| Primary, 1^st^ cycle | 5 (25) |
| Primary, 2^nd^ cycle | 3 (15) |
| Primary, 3^rd^ cycle | 7 (35) |
| Secondary education | 2 (10) |
| Higher education, bachelor | 0 (0) |
| Higher education, graduation | 3 (15) |
| Higher education, postgraduate studies | 0 (0) |
| Higher education, masters | 0 (0) |
| Higher education, PhD | 0 (0) |
| **Main occupation**, n (%) |  |
| Works on its own | 4 (20) |
| Works for others | 11 (55) |
| Student | 0 (0) |
| Doing military service | 0 (0) |
| Homemaker | 0 (0) |
| Retired | 4 (20) |
| Unemployed | 1 (5) |
| **Profession**, n (%) |  |
| Has no job | 0 (0) |
| Job | 20 (100) |
| **Professional sector**, n (%) |  |
| Primary sector | 0 (0) |
| Secondary sector | 1 (5) |
| Tertiary sector | 19 (95) |
| **Health care beneficiary** (multi response), n (%) |  |
| ADSE (State Health Service Assistance) | 1 (5) |
| SSMJ (Justice Ministry Service) | 0 (0) |
| IASFA (Institute of Social Action of the Armed Forces) | 0 (0) |
| SAD/PSP (PSP Disease Assistance Services) | 1 (5) |
| SAD/GNR (GNR Disease Assistance Services) | 0 (0) |
| SAMS (Banking Operations Department) | 1 (5) |
| Private Health Insurance | 0 (0) |
| SNS (National Health Service) | 17 (85) |
| Other | 0 (0) |
| Does not know | 0 (0) |
| **Geographic distribution (NUTS II)**, n (%) |  |
| North | 20 (0) |
| Center | 0 (0) |
| Metropolitan area of Lisbon | 0 (0) |
| Alentejo | 0 (0) |
| Algarve | 0 (0) |

NUTS II: Nomenclature of Territorial Units for Statistical Purposes.
