# Supplementary material for: Patients’ Perspectives about Lifestyle Behaviors and Health in the Context of Family Medicine: A Cross-Sectional Study in Portugal
Source: Int J Environ Res Public Health. 2021 Mar 14;18(6):2981. doi: 10.3390/ijerph18062981 (PMC8001049; doi:10.3390/ijerph18062981)
Supplement: Supplementary file 1 [file ijerph-18-02981-s001.zip › Suplementary files_13.03.2021/Table S2_Sociodemographic data_PORDATA.docx]

| **Table S3.** Sociodemographic data of the Portuguese population, obtained from de PORDATA website, www.pordata.pt. | | |
| --- | --- | --- |
|  |  | |
| **Age (years), n (%)^(1)^** |  | |
| 20-24 | 582065 (5.5) | |
| 25-29 | 656076 (6.2) | |
| 30-34 | 773567 (7.3) | |
| 35-39 | 824683 (7.8) | |
| 40-44 | 773098 (7.3) | |
| 45-49 | 770294 (7.3) | |
| 50-54 | 722360 (6.8) | |
| 55-59 | 677651 (6.4) | |
| 60-64 | 634741 (6.0) | |
| 65-69 | 551701 (5.2) | |
| 70-74 | 496438 (4.7) | |
| ≥75 | 961925 (9.1) | |
| **Gender, n (%)^(1)^** |  | |
| Male | 5046600 (47.8) | |
| Female | 5515578 (52.2) | |
| **Marital status, n (%)^(1)^** |  | |
| Not married | 4272977 (40.5) | |
| Married | 4924870 (46.6) | |
| Divorced | 593667 (5.6) | |
| Widowed | 770664 (7.3) | |
| **Higher educational level completed (of the resident population with ≥15 years), n in thousands (%)^(2)^** | | |
| None | 596.0 (6.7) | |
| Elementary school - 1^st^ cycle (4th year) | 1966.3 (22.2) | |
| Elementary school - 2^nd^ cycle (6th full year) | | 933.7 (10.5) |
| Elementary school - 3^rd^ cycle (9th full year) | | 1766.4 (20.0) |
| High school (12^th^ year) | 1934.5 (21.9) | |
| Higher Education | 1655.0 (18.7) | |
| **Main occupation, n in thousands (%)^(2)^** |  | |
| Works on its own | 789.7 (7.7) | |
| Work for others | 4056.5 (39.5) | |
| Student | 801.1 (7.8) | |
| Homemaker | 378.3 (3.7) | |
| Retired | 1.774.5 (17.3) | |
| Unemployed | 365.9 (3.6) | |
| **Professional sector, n in thousands (%)^(2)^** |  | |
| Primary | 294.2 (6.0) | |
| Secondary | 1209.2 (24.8) | |
| Tertiary | 3363.3 (69.1) | |
| **Mainland residence area (NUTS II), n (%)^(2)^** | | |
| North | 3572583 (34.8) | |
| Center | 2216569 (21.5) | |
| Lisbon | 2846332 (27.7) | |
| Alentejo | 705478 (6.9) | |
| Algarve | 438864 (4.3) | |

^(1)^PORDATA 2011, N=10562178 (mainland and islands)

^(2)^PORDATA 2018, N=10276617 (mainland and islands)
