# Supplementary material for: Patients’ Perspectives about Lifestyle Behaviors and Health in the Context of Family Medicine: A Cross-Sectional Study in Portugal
Source: Int J Environ Res Public Health. 2021 Mar 14;18(6):2981. doi: 10.3390/ijerph18062981 (PMC8001049; doi:10.3390/ijerph18062981)
Supplement: Supplementary file 1 [file ijerph-18-02981-s001.zip › Suplementary files_13.03.2021/Table S3_Comparison between levels of agreement of personal beliefs and personal self-report realities.docx]

**Table S4.** Comparison between levels of agreement of personal beliefs and personal self-report realities, for each lifestyle intervention.

|  | **Personal belief about**  **[95%CI]** | **Personal behavior self-assessment**  **[95%CI]** | **p-value** | **Level of importance given to the family doctor, to address**  **[95%CI]** | **If in the personal case, was usual or not, the family doctor asks/advises**  **[95%CI]** | **p-value** |
| --- | --- | --- | --- | --- | --- | --- |
| **Diet** | 5 [5; 5] | 4 [4; 4] | <0.001^*^ | 5 [5; 5] | 4 [3; 4] | <0.001^*^ |
| **Physical activity** | 5 [5; 5] | 3 [2; 3] | <0.001^*^ | 4.5 [4; 5] | 3 [3; 3] | <0.001^*^ |
| **Alcohol intake** | 5 [5; 5] | 5 [5; 5] ^#^ | <0.001^*^ | 4 [4; 4] | 2 [2; 2] | <0.001^*^ |
| **Tobacco use** | 5 [5; 5] | 5 [5; 5] ^#^ | <0.001^*^ | 4 [4; 4] | 2 [2; 3] | <0.001^*^ |
| **Illicit drugs** | 5 [5; 5] | 5 [5; 5] | 1.000 | 3 [3; 4] | 1 [1; 1] | <0.001^*^ |
| **Sleep habits** | 5 [5; 5] | 4 [4; 4] | <0.001^*^ | 5 [5; 5] | 3 [3; 4] | <0.001^*^ |
| **Screen activities** | 5 [5; 5] | 4 [4; 4] ^#^ | <0.001^*^ | 4 [3; 4] | 1 [1; 1] | <0.001^*^ |
| **Stress** | 5 [5; 5] | 4 [4; 4] ^#^ | <0.001^*^ | 5 [4; 5] | 3 [3; 3] | <0.001^*^ |
| **Sedentarism** | 5 [5; 5] | 4 [4; 5] ^#^ | <0.001^*^ | 5 [4; 5] | 3 [3; 3] | <0.001^*^ |

^*^Significant at 5% (Wilcoxon test). ^#^Inversion was performed in order to facilitate the comparison between the variables.
