# Supplementary material for: Patients’ Perspectives about Lifestyle Behaviors and Health in the Context of Family Medicine: A Cross-Sectional Study in Portugal
Source: Int J Environ Res Public Health. 2021 Mar 14;18(6):2981. doi: 10.3390/ijerph18062981 (PMC8001049; doi:10.3390/ijerph18062981)
Supplement: Supplementary file 1 [file ijerph-18-02981-s001.zip › Suplementary files_13.03.2021/Table S4_Characterization of lifestyle patterns of participants.docx]

**Table S2.** Characterization of lifestyle patterns of participants.

| **Body mass index (n=891)**, n (%) |  |
| --- | --- |
| Normal | 414 (46.6) |
| Overweight | 358 (40.2) |
| Obesity | 119 (13.4) |
| **Diet**, n (%) | |
| Number of **main meals** per day, 𝑥̅±sd | 2.7 ± 0.48 |
| 1 | 13 (1.4) |
| 2 | 221 (24.6) |
| 3 | 664 (73.8) |
| 4 | 2 (0.2) |
|  |  |
| Number of **fruit portions** per day (n=887), 𝑥̅±sd | 1.3 ± 0.6 |
| 1 | 645 (72.9) |
| 2 | 208 (23.5) |
| 3 | 22 (2.5) |
| 4 | 5 (0.6) |
| 5 | 4 (0.5) |
| 6 | 1 (0.1) |
|  |  |
| Number of **vegetables/salads portions** per day (n=885), 𝑥̅±sd | 1.8 ± 1.1 |
| 1 | 452 (51.0) |
| 2 | 273 (30.8) |
| 3 | 115 (13.0) |
| 4 | 31 (3.5) |
| 5 | 9 (1.0) |
| 6 | 2 (0.2) |
| 7 | 2 (0.2) |
|  |  |
| **Healthy diet** | 126 (14.0) |
|  |  |
| **Physical activity**, n (%) |  |
| **Daily predominant activity** |  |
| Sitting or standing in activities involving a slight physical effort | 342 (38.0) |
| On move or on tasks involving a moderate physical effort | 382 (42.4) |
| In heavy or physically demanding jobs | 73 (8.1) |
| None of these activities | 103 (11.4) |
| **Average walking time** of those who walk at least 10 minutes straight, per day (n=695) |  |
| Less than 10 minutes | 57 (8.2) |
| 10 to 29 minutes | 367 (52.8) |
| 30 to 59 minutes | 183 (26.3) |
| 1 hour to less than 2 hours | 31 (4.5) |
| 2 hours to less than 3 hours | 13 (1.9) |
| ≥3 hours | 44 (6.3) |
| **Average physical activity time** of those who practice at least 10 minutes straight, per day (n=406) |  |
| 10 to 29 minutes | 108 (26.6) |
| 30 to 59 minutes | 167 (41.1) |
| 1 hour to less than 2 hours | 99 (24.4) |
| 2 hours to less than 3 hours | 19 (4.7) |
| ≥3 hours | 13 (3.2) |
|  |  |
| **Regular physical activity** | 541 (60.1) |
|  |  |
| **Alcohol intake**, n (%) |  |
| **Frequency of alcohol consumption** |  |
| Every day or almost every day | 184 (20.4) |
| 5 to 6 days a week | 27 (3.0) |
| 3 to 4 days a week | 35 (3.9) |
| 1 to 2 days a week | 158 (17.6) |
| 2 to 3 days a month | 54 (6.0) |
| Once a month | 58 (6.4) |
| Less than once a month | 81 (9.0) |
| No drinking in the last 12 months for stopping alcohol intake | 152 (16.9) |
| Never drank or just occasionally for taste | 151 (16.8) |
| **Number of drinks** of frequent drinkers, per day (n= 297) |  |
| 1 | 162 (54.5) |
| 2 | 100 (33.7) |
| 3 | 17 (5.7) |
| 4 to 5 | 14 (4.7) |
| 6 to 9 | 4 (1.3) |
| From those who drink (n=597), how often **drank ≥6 drinks** |  |
| Every day or almost every day | 6 (1.0) |
| 5 to 6 days a week | 3 (0.5) |
| 3 to 4 days a week | 4 (0.7) |
| 1 to 2 days a week | 17 (2.8) |
| 2 to 3 days a month | 18 (3.0) |
| Once a month | 42 (7.0) |
| Less than once a month | 127 (21.3) |
| Never in the last 12 months | 190 (31.8) |
| Never in life | 190 (31.8) |
|  |  |
| **Adequate alcohol intake** | 649 (72.1) |
|  |  |
| **Tobacco use**, n (%) |  |
| yes | 231 (25.7) |
| no | 669 (74.3) |
| Never smoked | 511 (76.4) |
| **Smokers** (n=231), do it |  |
| Daily | 224 (97.0) |
| Occasionally | 7 (3.0) |
| Age of starting, those who smoke daily (n=224), 𝑥̅±sd, min, máx | 16.6 ± 3.9, 8, 50 |
|  |  |
| **Non-smokers** (n=669), frequency of being indoors with tobacco smoke: |  |
| Daily | 16 (2.4) |
| Occasionally | 125 (18.7) |
|  |  |
| **Non-smokers** | 669 (74.3) |
|  |  |
| **Illicit drugs**, n (%) |  |
| yes | 12 (1.3) |
| no | 888 (98.7) |
| **Those who consume** (n=12), do it |  |
| Daily | 7 (58.3) |
| Occasionally | 5 (41.7) |
| Age of starting, those who consume daily (n=7), 𝑥̅±sd, min, máx | 18.4± 3.8, 14, 26 |
| Those who consume, number of consumes per week, 𝑥̅±sd, min, máx | 5.3 ± 3.7, 1, 14 |
|  |  |
| **Do not consume illicit drugs** | 888 (98.7) |
|  |  |
| **Sleep habits**, n (%) |  |
| Frequency of **sleep problems** (difficulty falling asleep, slept poorly or overslept), in the last 2 weeks |  |
| Every day | 73 (8.1) |
| Most days | 89 (9.9) |
| Some days | 138 (15.3) |
| A few days | 155 (17.2) |
| Never | 445 (49.4) |
| Frequency of **need to take medication**, in the last 2 weeks |  |
| Every day | 71 (7.9) |
| Most days | 13 (1.4) |
| Some days | 30 (3.3) |
| A few days | 45 (5.0) |
| Never | 741 (82.3) |
| Frequency of a **restfull sleep**, in the last 2 weeks |  |
| Every day | 207 (23.0) |
| Most days | 347 (38.6) |
| Some days | 145 (16.1) |
| A few days | 118 (13.1) |
| Never | 83 (9.2) |
|  |  |
| **Good quality sleep** | 493 (54.8) |
|  |  |
| **Screen activities**, n (%) |  |
| **Number of days per week with screen activities** |  |
| 0 | 3 (0.3) |
| 1 | 6 (0.7) |
| 2 | 7 (0.8) |
| 3 | 7 (0.8) |
| 4 | 2 (0.2) |
| 5 | 16 (1.8) |
| 6 | 13 (1.4) |
| 7 | 846 (94.0) |
| **Average screen activity time** per day, (n= 897) |  |
| Less than 10 minutes | 9 (1.0) |
| 10 to 29 minutes | 46 (5.1) |
| 30 to 59 minutes | 125 (13.9) |
| 1 hour to less than 2 hours | 227 (25.3) |
| 2 hours to less than 3 hours | 171 (19.1) |
| ≥3 hours | 319 (35.6) |
| **Device** for screen activity |  |
| Television | 508 (56.6) |
| Computer | 166 (18.5) |
| Mobile phone | 223 (24.9) |
| Game consoles | 0 (0) |
|  |  |
| **Adequate screen activities** | 586 (64.6) |
|  |  |
| **Stress**, n (%) | |
| The frequency you **did less than wanted at work, due to anxiety**, in the last 2 weeks |  |
| Every day | 3 (0.3) |
| Most days | 12 (1.3) |
| Some days | 53 (5.9) |
| A few days | 132 (14.7) |
| Never | 700 (77.8) |
| **Calmness** frequency, in the last 2 weeks |  |
| Every day | 241 (26.8) |
| Most days | 409 (45.4) |
| Some days | 121 (13.4) |
| A few days | 97 (10.8) |
| Never | 32 (3.6) |
| **Anxiety felling** frequency, in the last 2 weeks |  |
| Every day | 36 (4.0) |
| Most days | 71 (7.9) |
| Some days | 139 (15.4) |
| A few days | 273 (30.3) |
| Never | 381 (42.3) |
| Frequency of **need to take medication**, in the last 2 weeks |  |
| Every day | 36 (4.0) |
| Most days | 17 (1.9) |
| Some days | 28 (3.1) |
| A few days | 31 (3.4) |
| Never | 788 (87.6) |
|  |  |
| **Adequate stress level** | 584 (64.9) |
